# CochleRob: Parallel-Serial Robot to Position a Magnetic Actuator around a Patient’s Head for Intracochlear Microrobot Navigation

**DOI:** 10.3390/s23062973

**Published:** 2023-03-09

**Authors:** Housseyne Nadour, Alexis Bozorg Grayeli, Gérard Poisson, Karim Belharet

**Affiliations:** 1 Centre National de la Recherche Scientifique (CNRS), GIPSA-Lab, École Doctorale Électronique, Électrotechnique, Automatique, Traitement du Signal (ED EEATS), 38100 Grenoble, France; 2 Department of Otolaryngology-Head and Neck Surgery, Dijon University Hospital, 21000 Dijon, France; 3 CNRS UMR 6306 Le2i Research Laboratory, 21078 Dijon, France; 4 PRISME EA 4229, Université d’Orléans, 45100 Orléans, France; 5 PRISME EA 4229, JUNIA-HEI, 2 Allée Jean Vaillé, 36000 Châteauroux, France

**Keywords:** hearing loss, remote drug delivery, drug administration, cochlea, microrobots, magnetic actuator, medical robot, control

## Abstract

Our work introduces a new robotic solution named CochleRob, which is used for the administration of super-paramagnetic antiparticles as drug carriers into the human cochlea for the treatment of hearing loss caused by damaged cochlea. This novel robot architecture presents two key contributions. First, CochleRob has been designed to meet specifications pertaining to ear anatomy, including workspace, degrees of freedom, compactness, rigidity, and accuracy. The first objective was to develop a safer mathod to administer drugs to the cochlea without the need for catheter or CI insertion. Secondly, we aimed at developing and validating the mathemathical models, including forward, inverse, and dynamic models, to support the robot function. Our work provides a promising solution for drug administration into the inner ear.

## 1. Introduction

The treatment of hearing loss due to inner ear damage has been a rapidly growing area of research and clinical trials in recent decades [1]. Many approaches have been proposed to treat different cochlear pathologies. Several robotic manipulators have been designed, and some of them have even been developed and tested in clinical studies [2,3,4,5,6].

The evolution of treatment approaches for cochlear pathologies has undergone two significant stages, moving from systemic administration to local administration. Based on the available information, researchers have proposed three methods of local administration: (1) transtympanic in situ drug administration method, which has limitations; (2) the catheter or CI implementation based method, which carries risk; and (3) the remote administration method, which is considered safe but requires a specialized robotic structure. With this paper, we aim to address this gap.

The shift from systemic to local administration in cochlear drug delivery is due to several limitations, such as the blood–cochlear barrier preventing drug diffusion [7,8] and the requirement for high doses of oral or intravenous drugs [9], leading to limited candidate drugs and treatment duration. Additionally, the requirement for long-term treatment and the presence of significant adverse side effects further discourage the use of systemic administration [10,11].

The transtympanic in situ drug administration method consists of injecting the drug into the middle ear cleft via the tympanic membrane under local anesthesia, delivering it to the cochlea through the round window membrane [12]. However, the amount delivered is poorly controlled by this method [13] and diffusion beyond the basal turn of the cochlea is limited due to negligible perilymphatic flux [14].

The CI surgery combined to residual hearing preservation has been successful [5,15,16,17]; however, there is a risk of damaging the cochlea’s internal structures during insertion of the electrode array [18]. This damage can occur from translocation of the array and friction on the cochlear lateral wall, and tip-folding of the array can cause trauma and device malfunction [19]. Efforts to reduce trauma through methods such as magnetic guidance have been attempted [20,21,22,23], but the placement of an array inside the cochlea is not considered a safe option for patients with inner ear diseases who do not require CI for hearing rehabilitation [14]. Developing robotic platforms specified for CI-based drug delivery is still a point of interest [24,25,26]. For more details about cochlear implants, the reader can refer to [27,28].

Our strategy, the remote administration method, consists of using therapeutic SPMNP (SuperParamagnetic NanoParticles) for drug delivery inside the cochlea [29]. This strategy does not require the insertion of any catheter or CI inside the cochlea, but only the particles, which considerably limits any casual damage to the cochlea. The particles will be controlled remotely to the cochlea using magnetic forces generated by our proposed magnetic actuator [30], positioned around the patient’s head. As a further development, we propose a novel manipulator robot to position the magnetic actuator around the patient’s head in this paper.

To outlines the plan of this article is as follows: The magnetic actuator’s operating principle and specifications are presented in Section 2. The design of the robotic system is described in Section 3. The forward and inverse kinematics models of the robot are explained in Section 4 and Section 5. The dynamic and space models are presented in Section 6 and Section 7. The joint control architecture is described in Section 8. The CochleRob manipulator prototype and experimental validation are detailed in Section 9. Conclusions and perspectives are provided in Section 10.

## 2. Design Specifications

### 2.1. Anatomical Specifications

**Figure 1:**
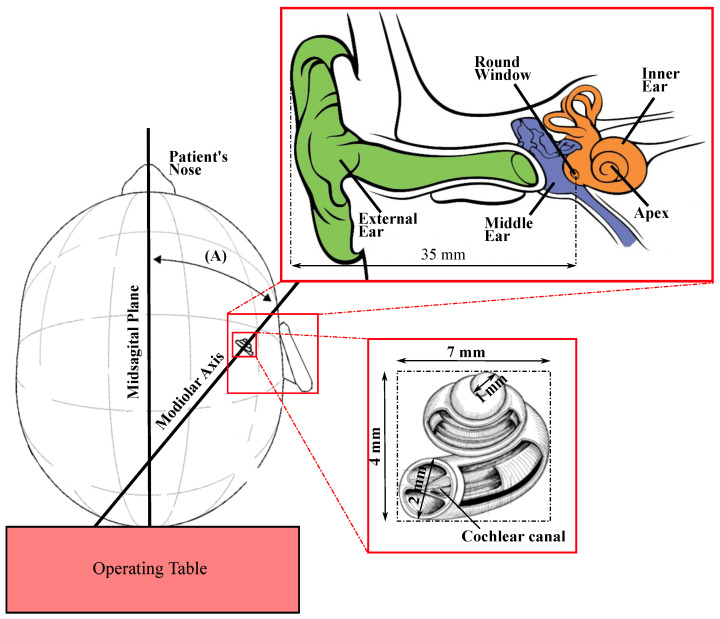
Position, orientation (A), and approximate dimensions of the cochlea [34].

The ear is composed of three parts: external ear, middle ear, and inner ear. The inner ear contains both the organ of hearing (cochlea) and the organ of balance (vestibular system). The cochlea, as it is viewed in Figure 1, is a set of membranous tubes, 31–33 mm. These tubes are coiled much like a snail shell to form two and a half turns around its axis [31]. There are two orifices at the external part of the cochlear bone in the middle ear, both of which are located at the base of the cochlea. The round window is a membranous opening in the bone within the scala tympani and the oval window, in the scala vestibuli. Perilymph is the fluid filling the scala tympani and vestibuli, and its volume in the human cochlea is about 70 µL. The height of the bony cochlea is approximately 4mm. The width of its basal coil, which is the largest, is about 7mm (see Figure 1). One can also note a slight inclination between turns, approximately 9 degrees between the basal and the middle turn and about 2.4 degrees between the middle and the apical turn [32]. Moreover, the cochlear spatial orientation has been studied in detail [33]. The modiolar axis of the cochlea has an average angle (A) of about 40 degrees with the Midsagittal plane. In addition, the cochlea’s axis is almost parallel to the horizontal plane of the skull since the lateral semicircular canal (LSC) forms an angle of 30 degrees forward and upward to the skull’s horizontal plane [22].

### 2.2. Magnetic Actuator Specifications

**Figure 2:**
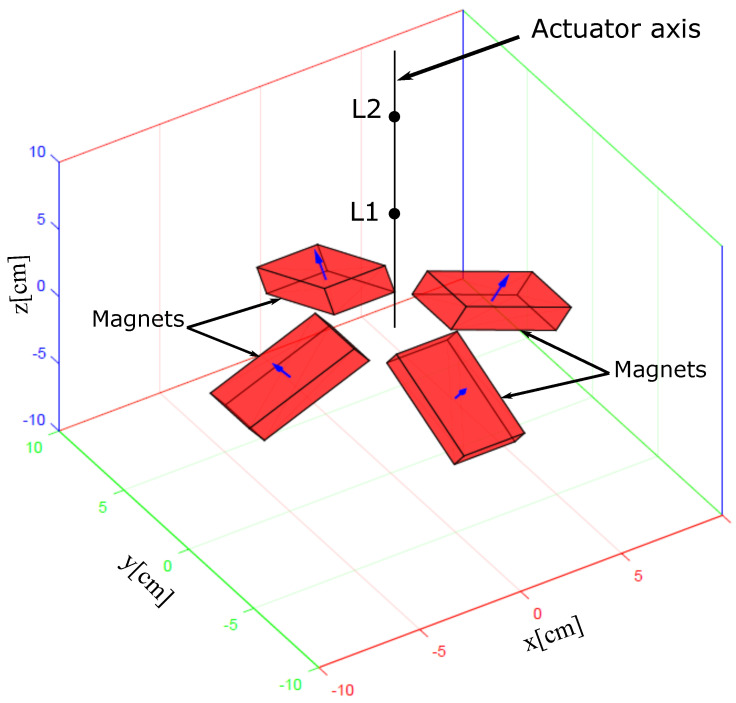
Four permanent magnets based actuator configuration.

**Figure 3:**
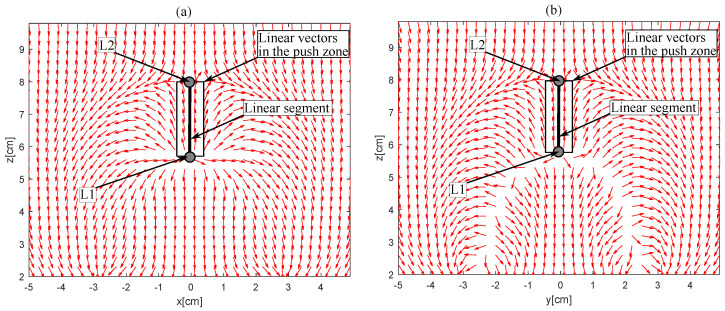
Magnetic force vectors generated by four-magnets actuator, which dimensions are 6 × 3 × 1.5 cm: (**a**) in the xz plane, (**b**) in the yz plane.

There are multiple solutions for propelling a microrobot in a viscous medium. The solution adopted here is to use a combination of permanent magnets [30,35]. Such a magnetic actuation system can generate magnetic fields that can induce effective forces to a magnetic device in a compact form-factor. The magnetic actutor proposed by our team [30] and presented in Figure 2 is studied to generate two Lagrangian points in the workspace, called $L_{1}$ (unstable point) and $L_{2}$ (stable point), Figure 3. We observe that, on the the actuator axis $L_{1}L_{2}$, the push force is generated between the two Lagrangian points and the pull force is generated elsewhere. This means that magnetic microrobot located around the point $L_{2}$ on the axis $L_{1}L_{2}$ are doomed to be pushed or pulled towards $L_{2}$. Hence, by positioning the segment $L_{1}L_{2}$ on a line connecting the magnetic microrobot and the desired position, the microrobot can be controlled in the viscous liquid of the inner ear, thanks to the attractive point $L_{2}$.

In a previous study conducted by our team [36], we introduced the guidance strategy for navigating a microrobot within the cochlea. This stragtegy aims at driving the magnatic microrobots (superparamagnetic nanoparticles) to the targeted cells in the inner ear. This strategy consists of four steps: (1). Image reconstruction, (2). Pre-planning, (3). Trajectory segmentation, and (4). Pushing/pulling force selection. The magnetic microrobots are encapsulated in a hydrogel and are deposited in contact with the round window membrane (entry point to the scala tympani in the middle ear, RWM, Figure 1).

The magnetic actuator is used to extract the microrobots from the gel. Then, it is used to control their movement through the RWM into the scala tympani and to the target area. The actuator offers the possibility of an open loop control to move the microrobots inside the cochlea from the RWM to the apex. The actuator’s axis must be aligned with the direction of the particle displacement in order to be able to push or pull particles in this correct direction. To perform the movement in different directions, we need to move the actuator around the head in the 3D space. This requires the use of a robotic manipulator, adapted to the spherical geometry of the workspace and the constraints related to the cochlea’s anatomy and the magnetic actuator.

## 3. Robot Design

### 3.1. Optimal Robot Degrees of Freedom and Workspace

The goal was to find the best possible combination of Degrees-of-Freedom (DoF) and workspace for this specific application. The operating principle of the magnetic actuator is the key element for defining the number of DoF. According to the previous actuator description (Section 2.2), in order to move the microrobots along the cochlear trajectory, the attractive Lagrangian point $L_{2}$ must move within this cochlear trajectory, while keeping the line $L_{1}L_{2}$ tangent on the trajectory at the point $L_{2}$. In that way, the microrobots will be adherent to $L_{2}$ or be in the neighborhood on the line $L_{1}L_{2}$. This causes them to instantly push towards L2 $L_{2}$: the microrobots always adhere to $L_{2}$.

**Figure 4:**
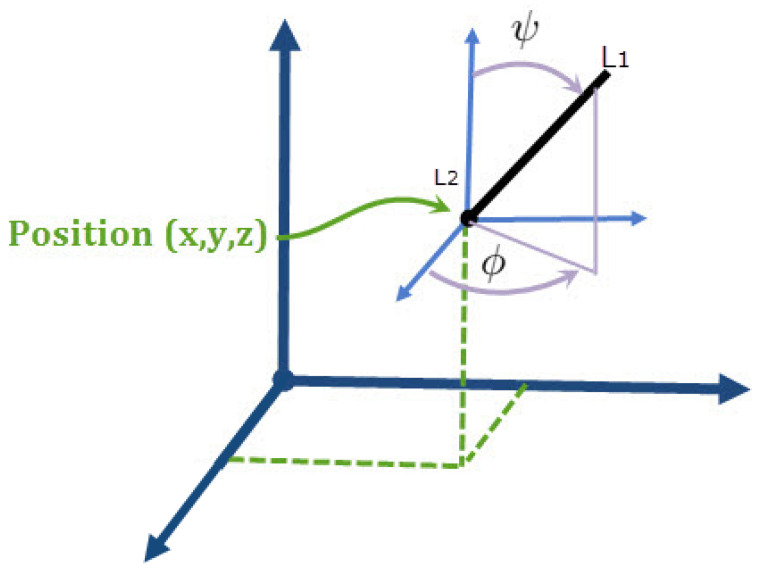
Positioning a segment ($L_{1}L_{2}$) in a 3D space.

**Figure 5:**
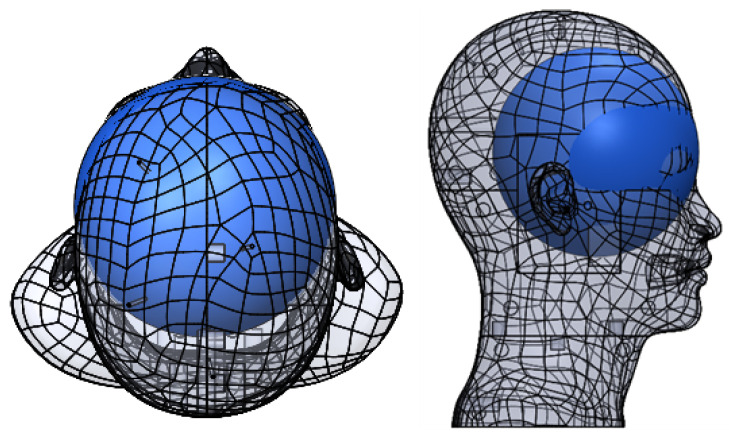
The sphere in which the point $L_{2}$ must move.

Positioning a point in space requires three DoF and orientating an axis needs two DoF, i.e., five DoF are necessary and sufficient to perform the desired task: three linear movements (*X*, *Y* and *Z*) and two rotational movements (ψ and ϕ) (Figure 4). The point, $L_{2}$, is supposed to be able to reach the top of the head, including the cochlea, on both sides. It should also be able to reach other important organs such as the brain and the eyes (for other medical purposes). Based on that, let the head be approximately a sphere (Figure 5) with a diameter equal to the bitemporal distance.

Note: the Lagrangian point $L_{2}$ is generally referred to as *P* in this paper, and some times as $P_{1}$ or $P_{2}$ when it is necessary to calculate in different frames.

### 3.2. From Specifications to the Shape of the Robot

For purpose of compactness, rigidity, and accuracy, we tried to look for a mechanical architecture that can fulfill all the aforementioned specifications as well as possible. Accuracy is mandatory because the point $L_{2}$ should travel through very small areas (like the cochlea) with high precision, while the structure must be rigid and able to bear significant weights since the magnetic actuator is around 1 kg. All of these factors must be considered together with the need for a compact platform.

The desired kinematic structure must be able to position and orientate the actuator in 3D space. The best way to have a compact structure that can perform rotational movements is to think about arcs: this kind of mechanism is called spherical Remote Center of Motion (RCM). See, for example [37,38].

The relevent types mentioned in [37] are Prism Robot for tele-echography, which is a spherical serial structure, and Basic Spherical Mechanism, which is a spherical parallel one that is used in [39]. However, these kinds of RCMs cannot be exploited in our case for reasons of mechanical constraints and rotation range. In [40], the author suggested a mechanism (called Otelo robot) for tele-echography. A similar mechanism of five-DoF is proposed in [40]. RCMs have been widely used in many works like [37], and it is used in this work for purpose of compactness.

As for purpose of rigidity and accuracy which are needed for moving the point $L_{2}$ in small area, we see that the Delta structure developed by Reymond Clavel in 1985 is more compatible. In fact, Alain Codourey, one of the most famous developers of this kind of robot, stated that the Delta robot possesses a number of advantages when compared to serial arms. The most important advantage is certainly the possibility to keep the motors fixed on the base, allowing for a large reduction of the active mobile mass of the robot structure [41]. Besides this feature, the Delta robot consists of three chains, each of which is composed of two segments rather than the three segments in the serial case (to create three degrees of freedom of translation). These two features makes the structure more compact. He also stated in the same article that another advantage of parallel robots is their higher rigidity: these features offer more accurate and much faster manipulations.

According to Clavel’s thesis [42], the Delta robot was developed in order to pick up and place light objects (20 g) at high dynamics. However, having three chains (in the Delta robot) connected to one point is meant in our case to increase the rigidity against the gravity, since the end-effector weight is around 1 kg and its speed is very low because its use is for medical purposes. In the same reference, it is stated that the Delta robot suffers from limited workspace (the price of rigidity and precision). This causes no trouble in our case, since the workspace needed to be reached is relatively small (see Figure 5).

### 3.3. Mechanical Design Description

**Figure 6:**
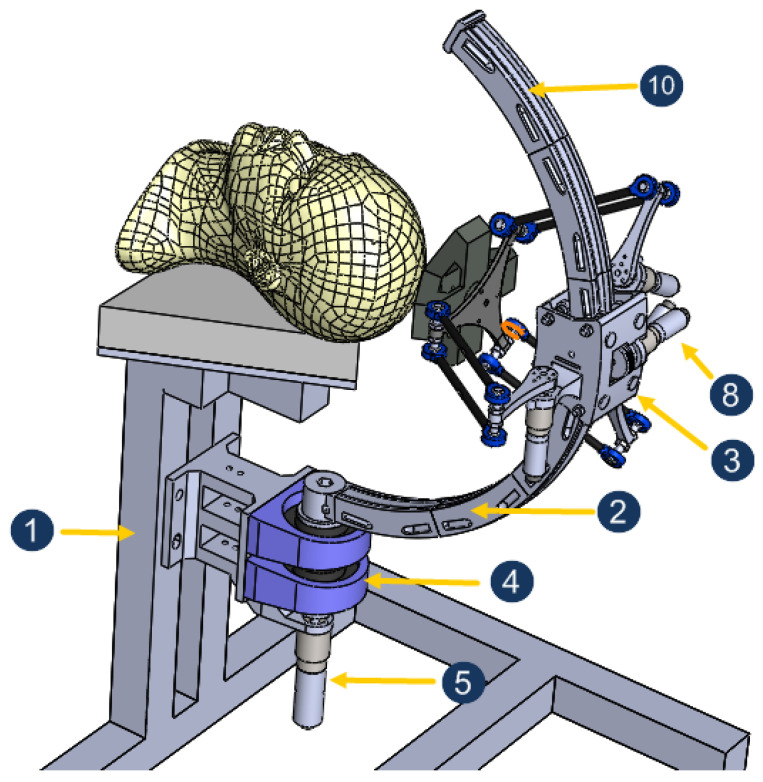
Mechanical design of the CochleRob manipulator.

**Figure 7:**
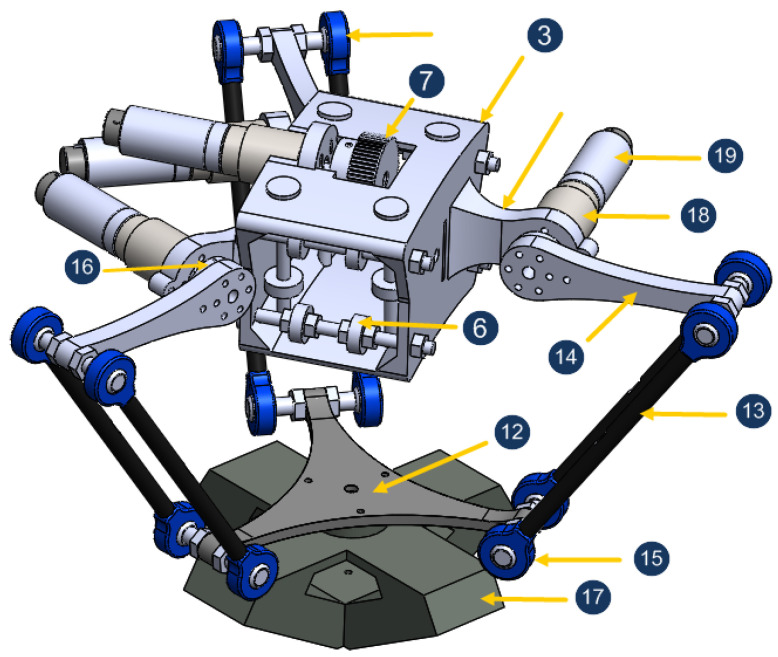
The parallel structure.

Figure 6 shows an assembly of different organs of the five-DoF robot. This robot is a hybrid combination of a spherical serial mechanism and a parallel one. It consists mainly of four parts: the support (1), the arc (the curved arm (2)), the slider (3), and the parallel structure (Figure 7). The arc is linked to the support by a without-friction ball-bearing articulation (4) and actuated by the motor (5) to generate the rotation ϕ. The slider can move smoothly on the arc thanks to a eight-balls-contact (6) and a pulley actuated by the motor (7). Rolling the pulley (8) on a belt (9) (pasted on the arc (10)) generates the rotation ψ. The slider itself represents a mobile base for the Delta structure, which consists mainly of three identical kinematic chains (11) linking the slider with a mobile board (12) (it is conventionally called nacelle in [42]). Each kinematic chain comprises an arm (13) and a forearm (14), joined together with the nacelle via ball joints (15). Each forearm is linked to its corresponding motor-gear axis via a hub (16). The fact that the arms have a parallelogram structure restricts the movement of the nacelle to pure translations with no rotation with respect to the slider. This makes it easy to control the magnetic actuator (17) linked to the nacelle. Finally, the gears (18) allows to reduce the motor speeds 231 times (3 × 7 × 11), so even with the energy lost in the gears, the torque is increased by more than 200 between the rotors (19) and the joints.

### 3.4. CochleRob Joints

**Figure 8:**
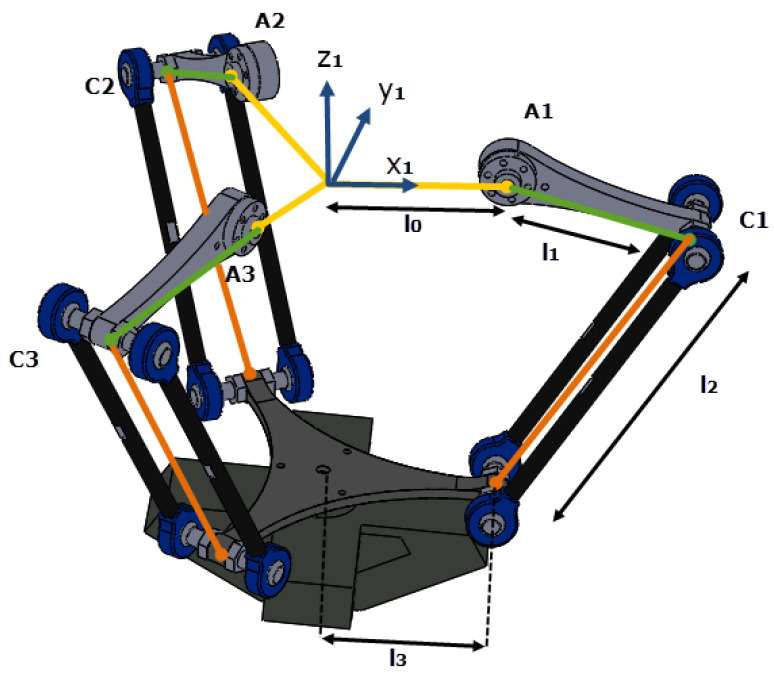
The kinematic chains of the delta part.

**Figure 9:**
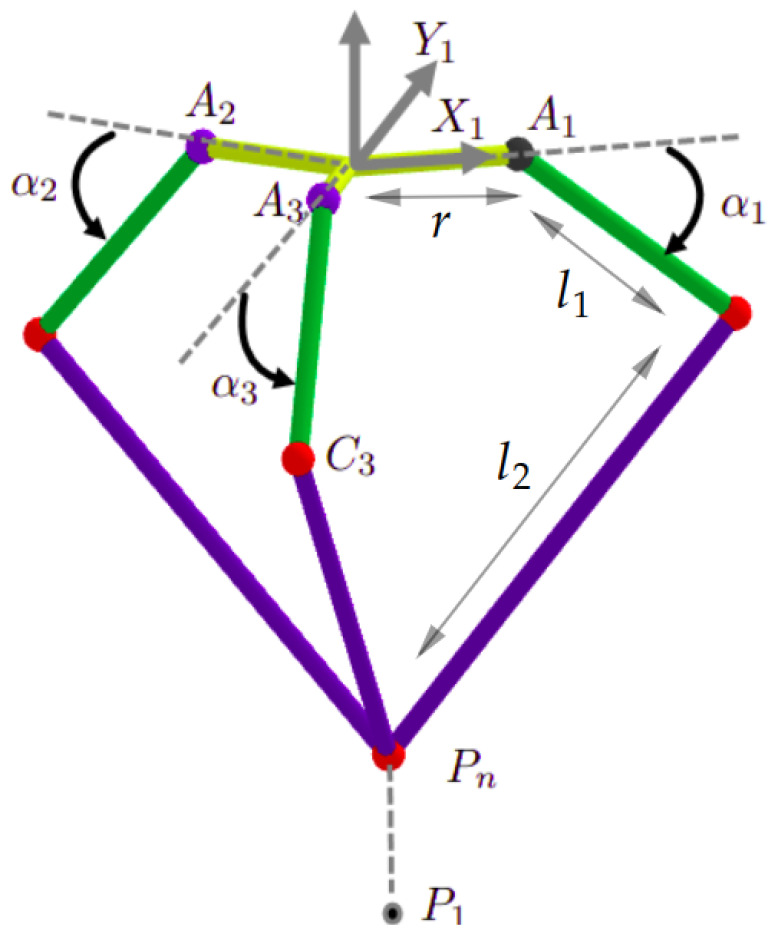
The simplification of the delta part, with $r=l_{0}-l_{3}$.

**Figure 10:**
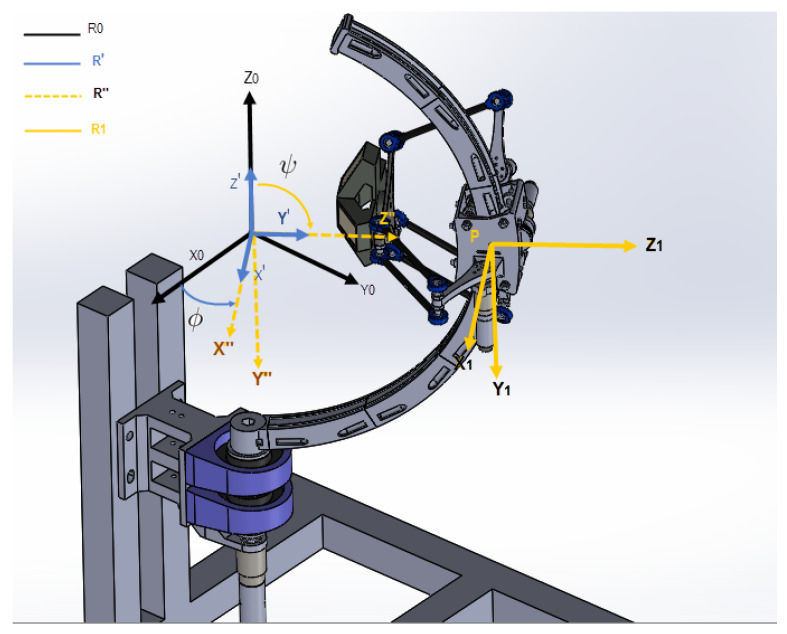
Transformations between frames.

Five joints are attributed to the robot for optimal number of degrees of freedom (DoF):

- The movement of the three kinematic chains of the delta structure is controlled by three joints, represented by the angles $\alpha_{1},\alpha_{2},$ and $\alpha_{3}$. These angles represent the angle between the forearm (14) and the relative horizontal plane. The different dimensions and joints of the delta part is illustrated in Figure 8. This part can be simplified if each chain (segments $l_{1}$ and $l_{2}$) is translated toward the axis $Oz_{1}$ by a distance $l_{0}$. The equivalent configuration is illustrated in Figure 9, with $r=l_{0}-l_{3}$.
- The fourth joint is represented with the angle ϕ. It is the rotation of the slider (3).
- The fifth joint is represented with the angle ψ. It is the rotation of the arc (2). See Figure 10.

## 4. Forward Kinematics Model

### 4.1. Modeling

**Figure 11:**
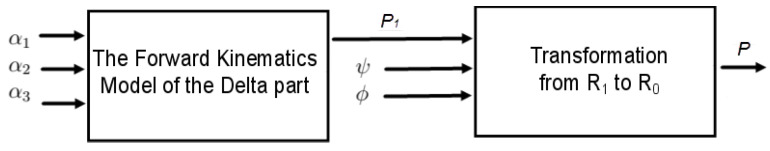
The forward kinematics model of the hybrid robot.

The Forward Kinematics Model (FKM) aims to calculate the end-effector coordinates (position and orientations) as a function of the joint variables of the mechanism (ψ, ϕ, $\alpha_{1}$, $\alpha_{2}$, $\alpha_{3}$), see Figure 11. The frames are assigned to the robot links in Figure 8 and Figure 10 so that the rotations angles of the end-effector are the same as the serial part joints angles (ψ, ϕ).

Having such a choice, we need just to find the end-effector position (X, Y, Z) as a function of ψ, ϕ, $\alpha_{1}$, $\alpha_{2}$, and $\alpha_{3}$, in order to establish the FKM. It is noticeable that the robot is composed of two main parts joined together in a serial chain:

- Serial part, in which the joints are ψ and ϕ.
- Parallel structure, for which the joints are $\alpha_{1}$, $\alpha_{2}$, and $\alpha_{3}$.

**Figure 12:**
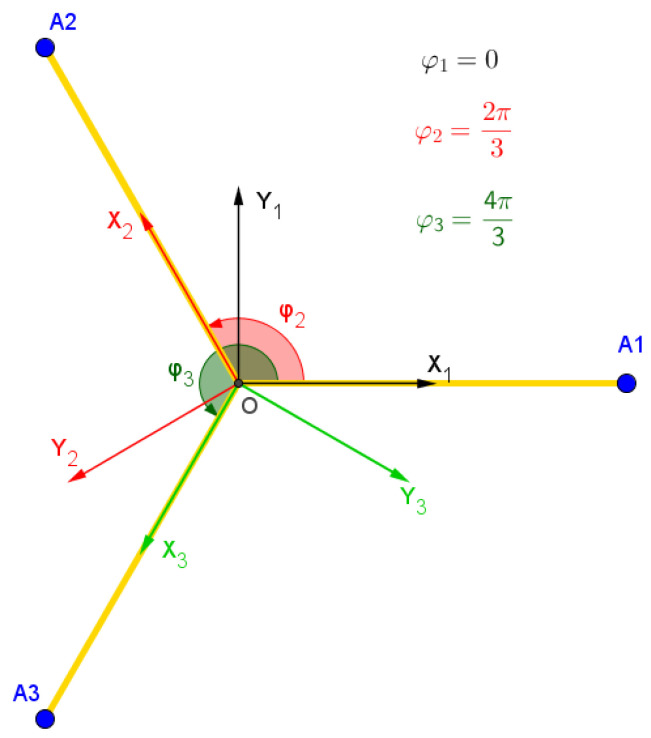
The different coordinates systems, $R_{i}$.

Let the following frames and points be defined as the following:

- $R_{0}=\left[{\vec{x}}_{0},{\vec{y}}_{0},{\vec{z}}_{0}\right]$ is the word frame, Figure 10.
- $R_{1}=\left[{\vec{x}}_{1},{\vec{y}}_{1},{\vec{z}}_{1}\right]$ is a mobile frame fixed on the slider (component (3) Figure 7), as indicated in Figure 8, Figure 9 and Figure 10.
- $R_{2}$ and $R_{3}$ are tow frames shifted from $R_{1}$ by rotations $\phi_{2}=\frac{2\pi }{3}$ and $\phi_{3}=\frac{4\pi }{3}$, respectively, around ${\vec{z}}_{1}$, Figure 12.
- $P_{n}=\left(\begin{array}{c} x_{n},y_{n},z_{n} \end{array}\right)$ is the position of the nacelle center (component 12 in Figure 7), expressed in frame $R_{1}$, Figure 8.
- $P=\left(\begin{array}{c} X,Y,Z \end{array}\right)$ is the end-effector position ($L_{2}$) expressed in $R_{0}$. It is the aim of this section.
- $P_{1}=\left(\begin{array}{c} x,y,z \end{array}\right)$ is the end-effector position ($L_{2}$), expressed in $R_{1}$.

In order to find the relationship between the joints angles and end-effector position *P*, we must first establish the expression of $P_{1}$ with respect to the moving frame $R_{1}$, as a function of $\alpha_{1}$, $\alpha_{2}$, and $\alpha_{3}$. In the second step, we project those calculated coordinates (x,y,z) into the steady frame $R_{0}$: it is a transformation from $R_{1}$ to $R_{0}$ based on ψ and ϕ, shown in Figure 11.

The rotation matrix from $R_{i}$ to $R_{1}$ is calculated as shown below (Equation (1)):

(1) $$A_{i}=\left(\begin{array}{ccc} \cos(\phi_{i}) & -\sin(\phi_{i}) & 0\\ \sin(\phi_{i}) & \cos(\phi_{i}) & 0\\ 0 & 0 & 1 \end{array}\right)$$

with: $\phi_{1}=0$, $\phi_{2}=\frac{2\pi }{3}$, $\phi_{3}=\frac{4\pi }{3}$.

The point $C_{i}$ has the following coordinates (Equation (2)):

(2) $$\begin{array}{c} C_{i}=A_{i}\cdot {\left(\begin{array}{c} r+l_{1}\cdot \cos\left(\alpha_{i}\right)\\ 0\\ -l_{1}\cdot \sin\left(\alpha_{i}\right) \end{array}\right)}_{R_{i}}={\left(\begin{array}{c} \left(r+l_{1}\cdot \cos\left(\alpha_{i}\right)\right)\cos\left(\phi_{i}\right)\\ \left(r+l_{1}\cdot \cos\left(\alpha_{i}\right)\right)\sin\left(\phi_{i}\right)\\ l_{1}\cdot \sin\left(\alpha_{i}\right) \end{array}\right)}_{R_{1}} \end{array}$$

Then, the vector $C_{i}P$ expressed in $R_{1}$, is obtained with (Equation (3)):

(3) $$C_{i}P=P-C_{i}={\left(\begin{array}{c} x-\left(r+l_{1}\cdot \cos\left(\alpha_{i}\right)\right)\cos\left(\phi_{i}\right)\\ y-\left(r+l_{1}\cdot \cos\left(\alpha_{i}\right)\right)\sin\left(\phi_{i}\right)\\ z+l_{1}\cdot \sin\left(\alpha_{i}\right) \end{array}\right)}_{R_{1}}$$

Equation (3) is used in the dynamic model. The method to calculate the coordinates $\left(x_{n},y_{n},z_{n}\right)$ of the nacelle position $P_{n}$ with respect to $R_{1}$ is explained in Clavel’s work [42], with the following equation (Equation (4)):

(4) $$\begin{array}{cc} z_{n} & =\frac{M-\sqrt{M^{2}-4\cdot L\cdot N}}{2\cdot L}\\ x_{n} & =\frac{H_{5}\cdot z+H_{4}}{H_{2}}\\ y_{n} & =\frac{H_{1}\cdot z+H_{3}}{H_{2}} \end{array}$$

with:

$$\begin{array}{cc} L & =1+\frac{H_{5}^{2}+H_{1}^{2}}{H_{2}^{2}}\\ M & =-2\cdot \frac{H_{5}\cdot H_{4}+H_{1}\cdot H_{3}}{H_{2}^{2}}+\frac{E_{1}\cdot H_{5}+F_{1}\cdot H_{1}}{H_{2}}+G_{1}\\ N & =D_{1}+\frac{H_{4}^{2}+H_{3}^{2}}{H_{2}^{2}}-\frac{E_{1}\cdot H_{4}+F_{1}\cdot H_{3}}{H_{2}} \end{array}$$

$$\begin{array}{cc} D_{1} & =-l_{2}^{2}+l_{1}^{2}+r^{2}+2\cdot r\cdot l_{1}\cdot \cos\left(\alpha_{1}\right)\\ D_{2} & =-l_{2}^{2}+l_{1}^{2}+r^{2}+2\cdot r\cdot l_{1}\cdot \cos\left(\alpha_{2}\right)\\ D_{3} & =-l_{2}^{2}+l_{1}^{2}+r^{2}+2\cdot r\cdot l_{1}\cdot \cos\left(\alpha_{3}\right)\\ E_{1} & =2\cdot \left(r+l_{1}\cdot \cos\left(\alpha_{1}\right)\right)\cdot \cos\left(\phi_{1}\right)\\ E_{2} & =2\cdot \left(r+l_{1}\cdot \cos\left(\alpha_{2}\right)\right)\cdot \cos\left(\phi_{2}\right)\\ E_{3} & =2\cdot \left(r+l_{1}\cdot \cos\left(\alpha_{3}\right)\right)\cdot \cos\left(\phi_{3}\right)\\ F_{1} & =2\cdot \left(r+l_{1}\cdot \cos\left(\alpha_{1}\right)\right)\cdot \sin\left(\phi_{1}\right)\\ F_{2} & =2\cdot \left(r+l_{1}\cdot \cos\left(\alpha_{3}\right)\right)\cdot \sin\left(\phi_{3}\right)\\ F_{3} & =2\cdot \left(r+l_{1}\cdot \cos\left(\alpha_{3}\right)\right)\cdot \sin\left(\phi_{3}\right)\\ G_{1} & =-2\cdot l_{1}\cdot \sin\left(\alpha_{1}\right)\\ G_{2} & =-2\cdot l_{1}\cdot \sin\left(\alpha_{2}\right)\\ G_{3} & =-2\cdot l_{1}\cdot \sin\left(\alpha_{3}\right)\\ H_{1} & =-\left(E_{3}-E_{1}\right)\cdot \left(G_{2}-G_{1}\right)+\left(G_{3}-G_{1}\right)\cdot \left(E_{2}-E_{1}\right)\\ H_{2} & =-\left(F_{3}-F_{1}\right)\cdot \left(E_{2}-E_{1}\right)+\left(E_{3}-E_{1}\right)\cdot \left(F_{2}-F_{1}\right)\\ H_{3} & =-\left(D_{3}-D_{1}\right)\cdot \left(E_{2}-E_{1}\right)+\left(E_{3}-E_{1}\right)\cdot \left(D_{2}-D_{1}\right)\\ H_{4} & =\left(D_{3}-D_{1}\right)\cdot \left(F_{2}-F_{1}\right)+\left(F_{3}-F_{1}\right)\cdot \left(D_{1}-D_{2}\right)\\ H_{5} & =-\left(G_{3}-G_{1}\right)\cdot \left(F_{2}-F_{1}\right)-\left(F_{3}-F_{1}\right)\cdot \left(G_{1}-G_{2}\right) \end{array}$$

Considering the distance *L* between the point $P_{n}$ and the attractive point $P_{1}$, we get the following expression, Equation (5):

(5) $$P_{1}=P_{n}+{\left(\begin{array}{c} 0\\ 0\\ -L \end{array}\right)}_{R_{1}}={\left(\begin{array}{c} x_{n}\\ y_{n}\\ z_{n}-L \end{array}\right)}_{R_{1}}$$

Finally, through a transformation (one translation and two rotations) from $R_{1}$ into frame $R_{0}$, we find the end effector position with respect to $R_{0}$:

(6) $$P=A_{0}^{\prime \prime }\cdot \left(P_{1}+\left(\begin{array}{c} 0\\ 0\\ R \end{array}\right)\right)=A_{0}^{\prime \prime }\cdot {\left(\begin{array}{c} x_{n}\\ y_{n}\\ z_{n}+R-L \end{array}\right)}_{R_{1}}$$

where *R* represents the arc radius, and

(7) $$A_{0}^{\prime \prime }=\left(\begin{array}{ccc} \cos(\phi ) & -\cos(\psi )\cdot \sin(\phi ) & -\sin(\psi )\cdot \sin(\phi )\\ \sin(\phi ) & \cos(\psi )\cdot \cos(\phi ) & \sin(\psi )\cdot \cos(\phi )\\ 0 & -\sin(\psi ) & \cos(\psi ) \end{array}\right)$$

### 4.2. Validation of the FKM Model

The aim of this validation is to compare our analytic FKM with the numerical FKM exported from SolidWorks to SimMechanics. For identical articular inputs, we compared the end-effector positions and orientations obtained by the two computational approaches (analytic and numerical models).

**Figure 13:**
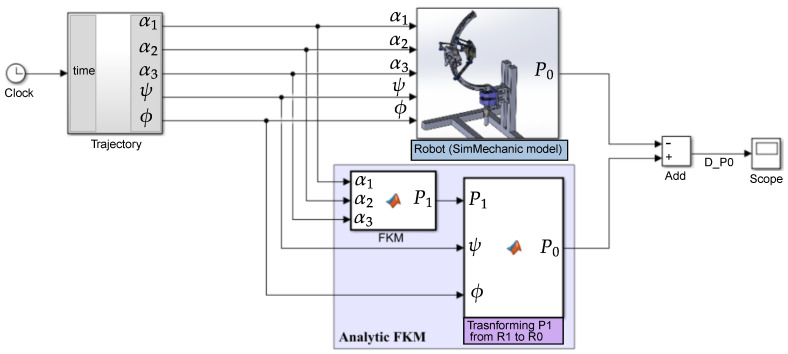
Simulink diagram to compare the analytic FKM model with the numerical model exported from SolidWorks to SimMechanics.

**Figure 14:**
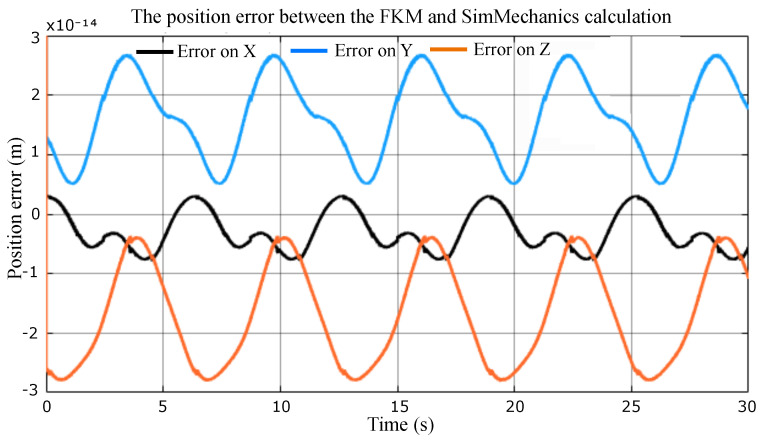
Comparison between the analytic FKM model and the numerical model exported from SolidWorks to SimMechanics.

Figure 13 shows the difference between two signals representing the end-effector position $P=\left(\begin{array}{c} X,Y,Z \end{array}\right)$ with respect to frame $R_{0}$. One signal is from the block named *Robot* representing the model imported from SolidWorks environment. The other comes from the block named Analytic FKM representing the model calculated in this section. Both diagrams receive their inputs from the same source; the result of the simulation is illustrated in Figure 14.

The simulation shows that the FKM and SolidWorks approaches give very similar results since the errors are null ($10^{-14}\approx 0$). The FKM elaborated in this section is thus validated.

## 5. Inverse Kinematics Model

### 5.1. Modeling

**Figure 15:**
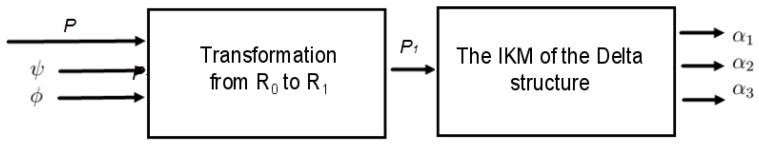
Inverse Kinematics Model of the hybrid robot.

The Inverse Kinematics Model (IKM) aims to calculate the joints angles $\alpha_{1}$, $\alpha_{2}$, $\alpha_{3}$, ψ, and ϕ as a function of rotation and position *P* of the end-effector, see Figure 15.

As mentioned previously, the end-effector rotations are the same as the serial part joint angles ψ and ϕ. Thus, the objective of this section is to calculate $\alpha_{1}$, $\alpha_{2}$, and $\alpha_{3}$ in terms of (X,Y,Z), ψ, and ϕ.

The transformation from *P* to $P_{1}$ can be calculated easily by following the opposite steps indicated in the previous section. A superior option would be to establish it in a straightforward manner from Equation (6):

(8) $$P_{1}={A_{0}^{\prime \prime }}^{t}\cdot P_{0}-{\left(\begin{array}{c} 0\\ 0\\ R \end{array}\right)}_{R_{1}}$$

Another backward step concluded from Equation (5):

(9) $$P=P_{1}+{\left(\begin{array}{c} 0\\ 0\\ L \end{array}\right)}_{R_{1}}={\left(\begin{array}{c} x\\ y\\ z+L \end{array}\right)}_{R_{1}}$$

Multiple formulations have been proposed in order to calculate the IKM of the Delta robot [42,43]. The one used in [42] consists of

(10) $$\alpha_{i}=\arctan\left(\frac{-2z+F_{i}}{-2r-S-Q_{i}\left(\frac{r}{l_{1}}-1\right)}\right)$$

where:

$$\begin{array}{cc} F_{i} & =\sqrt{4z^{2}+4r^{2}-S^{2}+Q_{i}^{2}\left(1-\frac{r^{2}}{l_{1}^{2}}\right)+Q_{i}\left(-2\frac{r\cdot S}{l_{1}}-4r\right)}\\ Q_{i} & =2\cdot x\cdot \cos\left(\phi_{i}\right)+2\cdot y\cdot \sin\left(\phi_{i}\right)\\ S & =\frac{1}{l_{1}}\left(-x^{2}-y^{2}-z^{2}+l_{2}^{2}-l_{1}^{2}-r^{2}\right) \end{array}$$

### 5.2. Numerical Validation of the IKM Model

**Figure 16:**
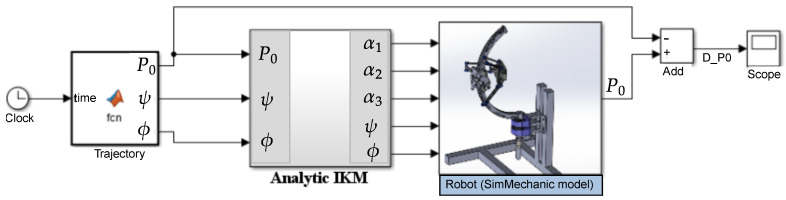
Simulink diagram to validate the IKM implicitly, using FKM.

Since there is no numerical IKM to compare with, we used the previous numerical (or analytical) FKM to validate our analytic IKM implicitly. Contrary to the previous validation, where we put the two models in parallel connection and compared their outputs, this time, we put our analytical IKM and the FKM in series as described in Figure 16. Eventually, if the IKM maps correctly from their inputs, i.e., the Cartesian coordinates (X,Y,Z,ϕ,θ) to the joints’ parameters $\left(\alpha_{1},\cdots ,\theta \right)$, the outputs of FKM (X,Y,Z) (the block named *Robot*) must be identical to the inputs of the analytic IKM (X,Y,Z).

**Figure 17:**
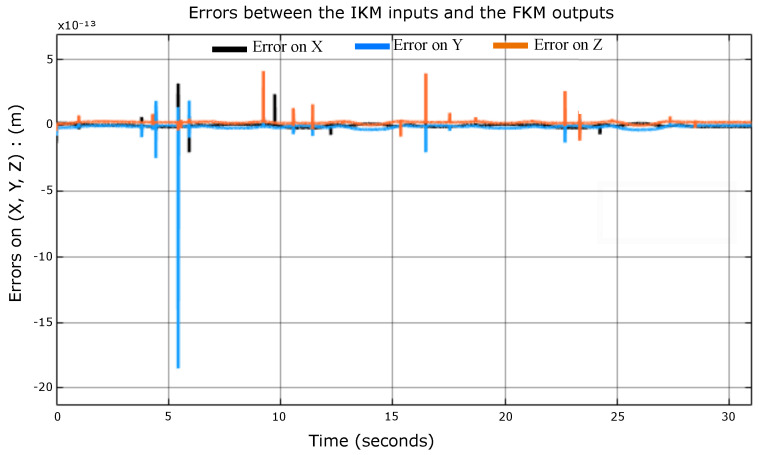
The error between the IKM inputs and the numerical FKM outputs.

The scope in Figure 17 shows the errors are null ($10^{-13}\approx 0$), which means that the IKM maps correctly.

## 6. The Dynamic Model

**Figure 18:**
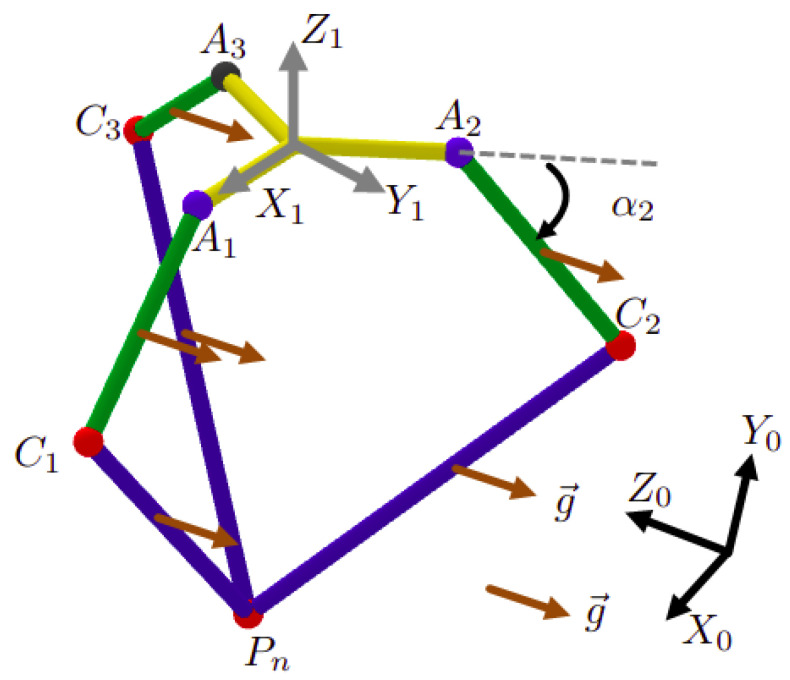
The gravity with respect to the Delta structure.

This section presents a dynamic model of the hybrid robot that defines the necessary torques, $T_{i}$, that must be applied by each motor *i* when the mechanism performs some desired task: see Figure 18. Obviously, the torque $T_{i}$, provided to the different kinematic chains, has the role to undo the gravity effects on the robot organs. It also must ensure that they travel through space within some desired acceleration as well: see Equation (11):

(11) $$T_{i}=T_{i_{g}}+T_{i_{a}}$$

Such that

- $T_{i}$ is the torque generated by motor *i* (electromagnetic torque).
- $T_{i_{g}}$ is the necessary torque provided by motor *i* to make the robot steady in front of the gravity affect: we call it the gravity torque.
- $T_{i_{a}}$ is the torque provided by each motor *i*, when the different organs perform specific accelerations in gravity-free space: we call it the acceleration torque.

As described previously, during the operation, the end effector travels through the space with a very low velocity. Moreover, its mass is very considerable (around 1 kg), which means the acceleration torque is negligible when compared to the gravity torque effect. In addition, the gear ratio (n=231) is also an important reason to ignore the acceleration torque of the different organs. Thus, the only dynamic that will be involved is the angular acceleration ($\ddot{\theta_{i}}$) of each motor rotor, since it is not affected by the gearbox reduction. That being said, the acceleration torque is reduced to $T_{i_{a}}=J_{i}\cdot \ddot{\theta_{i}}$. This fact allows us to have a very simplified and accurate model, rather than a very complicated one. Thus, the Equation (11) becomes Equation (12):

(12) $$T_{i}=T_{ia}+T_{ig}=J_{i}\cdot \ddot{\theta_{i}}+T_{ig}$$

The following study will establish the dynamic model of the Delta structure. Then, the dynamic model of the serial structure will be established.

### 6.1. The Dynamic Model of the Delta Structure

The main factor in this section is the gravity. With respect to the original base $R_{0}$, it is a vector that has one component, laid upon the z-axis:

(13) $$\vec{g}={\left(\begin{array}{c} 0,0,-9.81 \end{array}\right)}^{t}$$

Meanwhile, it is a mobile vector with respect to the Delta structure.

Having the gravity $g_{1}$ expressed in $R_{1}$ is more compatible to calculate the gravity torques $T_{i_{g}}$ for the Delta structure. $\vec{g_{1}}$ is the expression of the gravity in $R_{1}$:

(14) $$\vec{g_{1}}={A_{0}^{\prime \prime }}^{-1}\cdot \vec{g}$$

Let be the following denotations:

- $M_{1}$: the mass of the forearm $l_{1}$.
- $M_{2}$: the mass of the parallel sticks $l_{2}$.
- $M_{3}$: the mass of the nacelle.
- the center of mass of each parallel sticks is the midpoint of the segment $l_{2}$.
- the center of mass of each forearm is at a distance of $d_{1}$ from the corresponding joint $A_{i}$.

**Figure 19:**
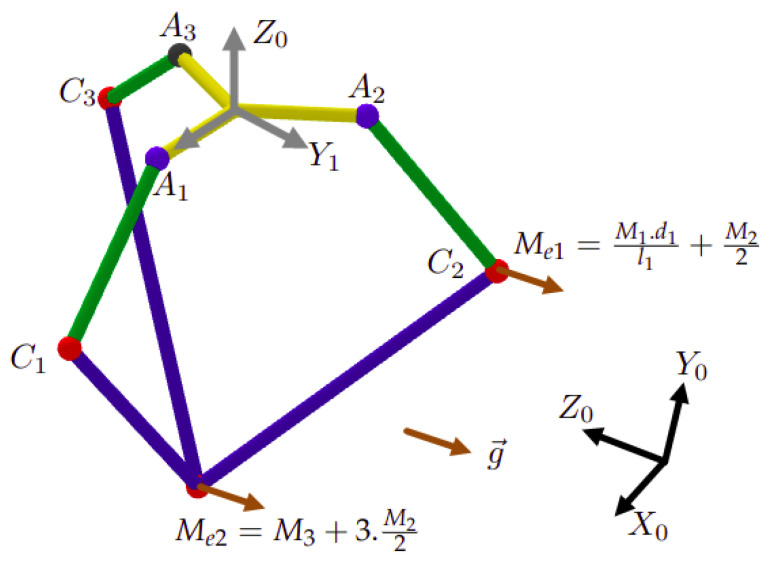
Equivalent masses.

Having three masses $M_{1}$, $M_{2}$, and $M_{3}$ on each kinematic chain (Figure 18) is equivalent to having two masses $M_{e_{1}}$ and $M_{e_{2}}$ linked to the corresponding elbow $C_{i}$ and the point *P*, respectively, (roughly speaking, the configuration Figure 18 is equivalent to the configuration Figure 19), with

$$M_{e1}=\frac{M_{1}\cdot d_{1}}{l_{1}}+\frac{M_{2}}{2}\;\mathrm{and}\;M_{e2}=M_{3}+3\cdot \frac{M_{2}}{2}$$

The gravitational forces (expressed in $R_{1}$) of those two masses are

(15) $$\left\{\begin{array}{c} \vec{N_{i}}=\vec{g_{1}}\cdot M_{e_{1}},\mathrm{with}:i=1,2,3.\\ \vec{Z}=\vec{g_{1}}\cdot M_{e_{2}} \end{array}\right.$$

**Figure 20:**
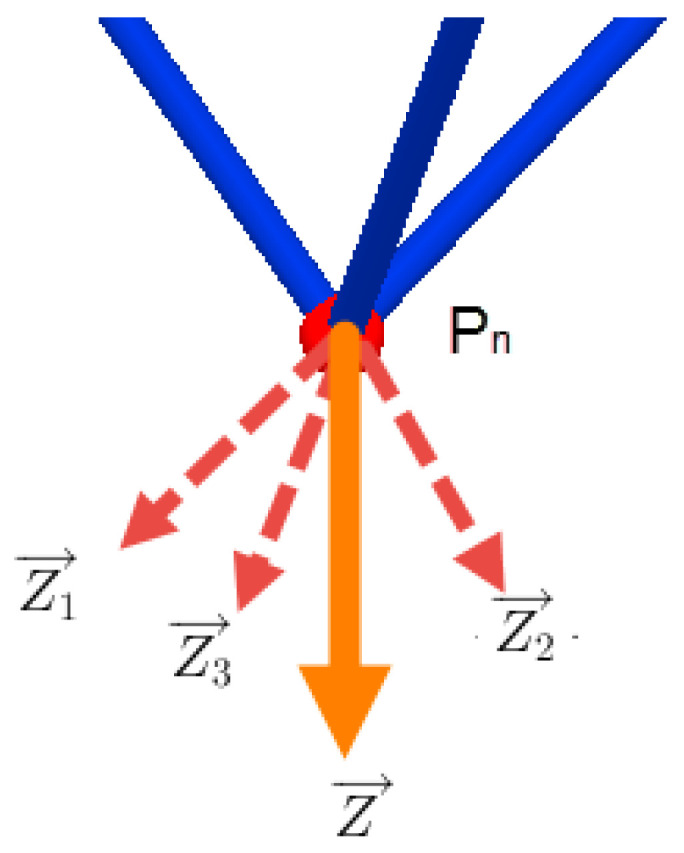
Component of Z with respect to the parallel segments.

The force $\vec{N_{i}}$ is applied on the elbow $C_{i}$, while $\vec{Z}$ is applied on the point *P*. It is composed of three components laid on the parallel segments. shown in Figure 20:

(16) $$\vec{Z}=\vec{Z_{u_{1}}}+\vec{Z_{u_{2}}}+\vec{Z_{u_{3}}}$$

given that:

(17) $$\left\{\begin{array}{c} \vec{Z_{u_{1}}}=Z_{u_{1}}\cdot \vec{u_{1}}\\ \vec{Z_{u_{2}}}=Z_{u_{2}}\cdot \vec{u_{2}}\\ \vec{Z_{u_{3}}}=Z_{u_{3}}\cdot \vec{u_{3}} \end{array}\right.$$

where $\vec{u_{1}}$, $\vec{u_{2}}$, and $\vec{u_{3}}$ are unit vectors expressed in $R_{1}$ and carried on the parallel segments ($l_{2}$), with

(18) $$\vec{u_{1}}=\frac{\vec{C_{1}P}}{C_{1}P},\vec{u_{2}}=\frac{\vec{C_{2}P}}{C_{2}P},\vec{u_{3}}=\frac{\vec{C_{3}P}}{C_{3}P}$$

with $C_{i}P$ is determined in Equation (3). Then, the Equation (16) becomes

(19) $$\vec{Z}=\left[\vec{u_{1}},\vec{u_{2}},\vec{u_{3}}\right]\left(\begin{array}{c} Z_{u_{1}}\\ Z_{u_{3}}\\ Z_{u_{3}} \end{array}\right)$$

Thus, the components of the force $\vec{Z}$ with respect to the unit base $\left[\vec{u_{1}}\;\vec{u_{2}}\;\vec{u_{3}}\right]$ are well determined:

(20) $$\left(\begin{array}{c} Z_{u_{1}}\\ Z_{u_{3}}\\ Z_{u_{3}} \end{array}\right)={\left[\vec{u_{1}},\vec{u_{2}},\vec{u_{3}}\right]}^{-1}\vec{Z}$$

**Figure 21:**
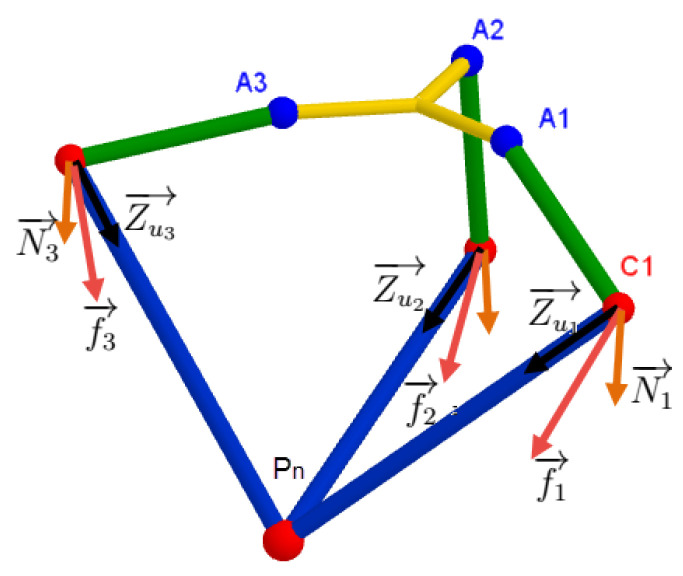
The forces applied on each elbow $C_{i}$.

As illustrated in (Figure 21), the total force applied on each elbow $C_{i}$ is

(21) $$\underset{\mathrm{expressed}\mathrm{in}R_{1}}{\underbrace{\vec{f_{i}}=Z_{u_{i}}.\vec{u_{i}}+\vec{N_{i}}}}$$

**Figure 22:**
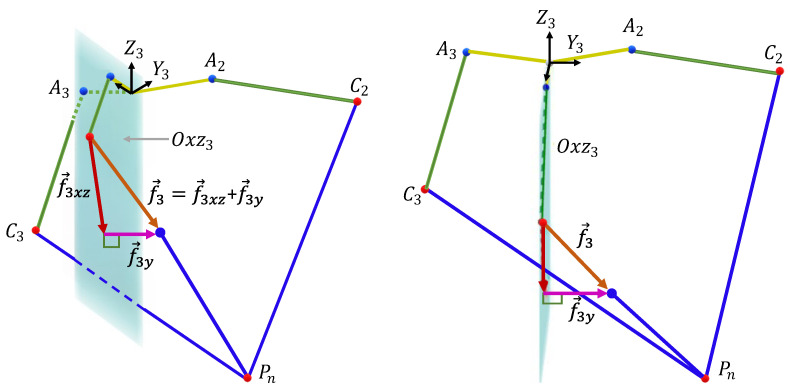
The projection of the force $f_{3}$ on the plane $Oxz_{3}$.

Each elbow *i* belongs to its associated base $R_{i}$. Expressing $\vec{f_{i}}$ in this base is convenient to next calculations. To do that, we multiply $\vec{f_{i}}$ by $A_{i}^{-1}=A_{i}^{T}$. This matrix is elaborated in Equation (1). Moreover, each forearm has only one freedom in the plane $Oxz_{i}$, meaning that the only component of $\vec{f_{i}}$ that has mechanical work is $f_{ixz}$. The component $f_{iy}$ has no effect: see Figure 22:

(22) $$\underset{\mathrm{expressed}\mathrm{in}R_{i}}{\underbrace{\vec{f_{ixz}}=\left(\begin{array}{ccccc} 1 & \; & 0 & \; & 0\\ 0 & \; & 0 & \; & 0\\ 0 & \; & 0 & \; & 1 \end{array}\right)\cdot A_{i}^{T}\cdot \vec{f_{i}}}}$$

Next, the torque due to the gravity applied on each forearm *i*, with respect to its corresponding joint, $A_{i}$, is

(23) $$\left(\begin{array}{c} 0,1,0 \end{array}\right)\cdot \left(\begin{array}{c} \vec{AC_{i}}\times \vec{f_{ixz}} \end{array}\right)$$

where:

(24) $$\vec{AC_{i}}={\left(\begin{array}{c} l_{1}\cdot \cos\left(\alpha_{i}\right)\\ 0\\ -l_{1}\cdot \sin\left(\alpha_{i}\right) \end{array}\right)}_{R_{i}}$$

Finally, the torque provided by each actuator *i* associated with the joint $A_{i}$ is

(25) $$T_{i_{g}}=-\left(\begin{array}{c} 0,1,0 \end{array}\right)\cdot \left(\begin{array}{c} \vec{AC_{i}}\times \vec{f_{ixz}} \end{array}\right)\cdot \frac{1}{231}$$

The dot product has appeared because the torque vector $\vec{AC_{i}}\times \vec{f_{ixz}}$ has no component on the plane $Oxz_{i}$, but rather, only one component on the y-axis that affects the forearm. The minus sign has been introduced because the torque provided to the joint *i* must oppose the gravity torque in order to prevent its effect on the elbow. The number 231 represents the gearbox ratio. By dividing by this ratio, we get the torque that must be generated by the motor *i*.

### 6.2. The Dynamic Model of the Serial Structure

The gravity has no effect on the fifth joint (ϕ). Then,

(26) $$T_{5g}=0$$

**Figure 23:**
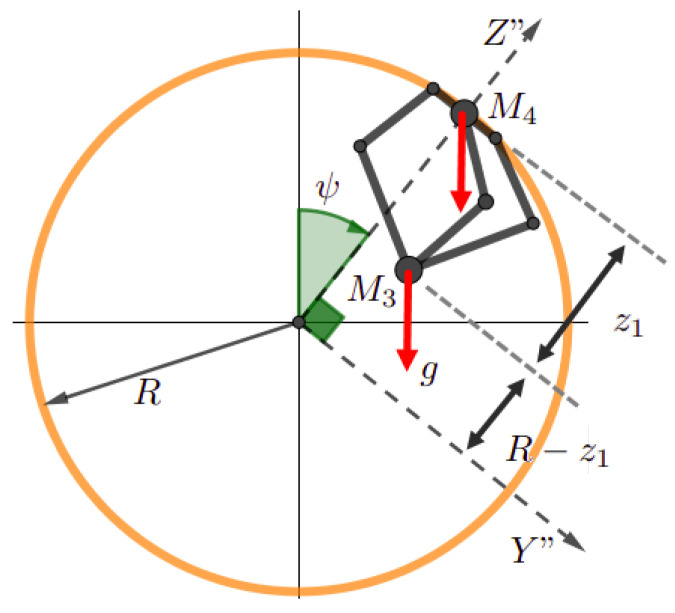
The substantial forces to generate $T_{4g}$.

It substantially affects the fourth actuator, associated to (ψ), due to the end-effector mass $M_{3}$ and the slider mass $M_{4}$, as is illustrated in Figure 23. The torque generated with respect to the point *O* is

(27) $$C=\left(\left(R-z_{n}\right)\cdot M_{3}+R\cdot M_{4}\right)\cdot \sin\left(\psi \right)$$

Although the masses of the kinematic chains are neglected, the torque due to these masses can be approximately introduced in the previous equation as following:

(28) $$\begin{array}{cc} C & =\left(\left(R-z_{n}\right)\cdot \left(M_{3}+3\cdot \frac{M_{1}+M_{2}}{2}\right)\right.+\left.R\cdot \left(M_{4}+3\cdot \frac{M_{1}+M_{2}}{2}\right)\right)\cdot \sin\left(\psi \right) \end{array}$$

Let $\psi^{\prime }$ be the rotation of the pulley (component (7) Figure 7), $C^{\prime }$ be the torque generated by the pulley, and *C* be the torque generated by a fictive actuator hypothetically placed at the center *O*. Then, the work provided by the pulley, $C^{\prime }.d\psi^{\prime }$, equals the work provided by the fictive actuator, C·dψ.

(29) $$C^{\prime }\cdot d\psi^{\prime }=C\cdot d\psi$$

**Figure 24:**
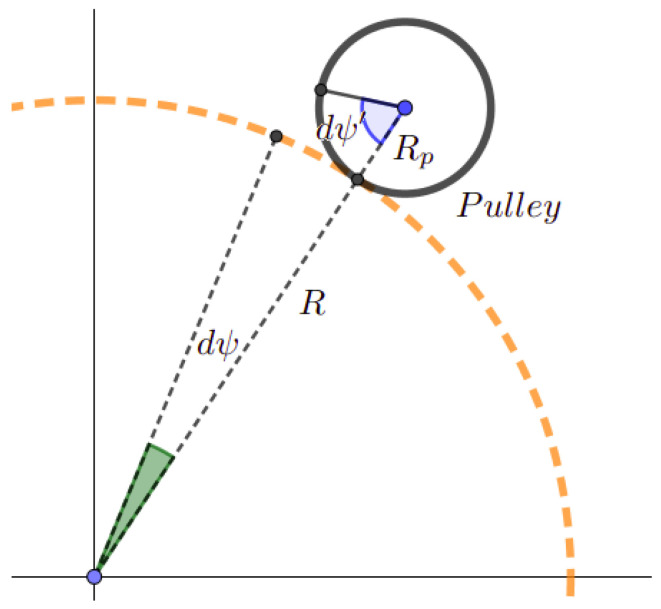
The relation between the pulley rotation and ψ.

Since there is no sliding between the pulley and the arc (as it is demonstrated in Figure 24),

(30) $$d\psi =d\psi^{\prime }\cdot \frac{R_{p}}{R}$$

where $R_{p}$ is the pulley radius and *R* is the arc radius (component (2) Figure 7).

From Equations (29) and (30),

(31) $$C^{\prime }=C\cdot \frac{R_{p}}{R}$$

By introducing the gearbox ratio of n=231, we get

(32) $$T_{4g}=\frac{C^{\prime }}{231}$$

Finally:

(33) $$T_{4g}=\sin\left(\psi \right)\frac{R_{p}}{231\cdot R}\cdot \left(\left(R-z_{n}\right)\cdot \left(M_{3}+3\cdot \frac{M_{1}+M_{2}}{2}\right)\right.+\left.R\cdot \left(M_{4}+3\cdot \frac{M_{1}+M_{2}}{2}\right)\right)$$

Note that the motors rotation angles have the following relations:

(34) $$\left\{\begin{array}{cc} \theta_{i} & =231\cdot \alpha_{i}\;\;\;\;\;\;\;;\;i=1,2,3.\\ \theta_{4} & =\frac{231\cdot R}{R_{p}}\cdot \psi \\ \theta_{5} & =231\cdot \phi \end{array}\right.$$

To conclude, the dynamic model is briefly described by the following equations:

(35) $$\left\{\begin{array}{c} T_{i\in \{1,2,3\}}=J_{i}\cdot \ddot{\theta_{i}}-\left(\begin{array}{c} 0,1,0 \end{array}\right).\left(\begin{array}{c} \vec{AC_{i}}\times \vec{f_{ixz}} \end{array}\right).\frac{1}{231}\\ T_{4}=J_{4}\cdot \ddot{\theta_{4}}+\sin\left(\psi \right)\frac{R_{p}}{231\cdot R}\cdot \left(\left(R-z_{n}\right)\cdot \left(M_{3}+3\cdot \frac{M_{1}+M_{2}}{2}\right)\right.+\left.R\cdot \left(M_{4}+3\cdot \frac{M_{1}+M_{2}}{2}\right)\right)\\ T_{5}=J_{5}\cdot \ddot{\theta_{5}} \end{array}\right.$$

### 6.3. Validation of the Dynamic Model

**Figure 25:**
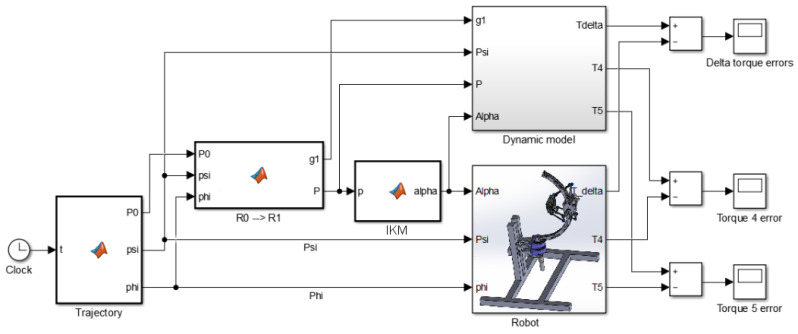
Simulink diagram to test the dynamic model.

**Figure 26:**
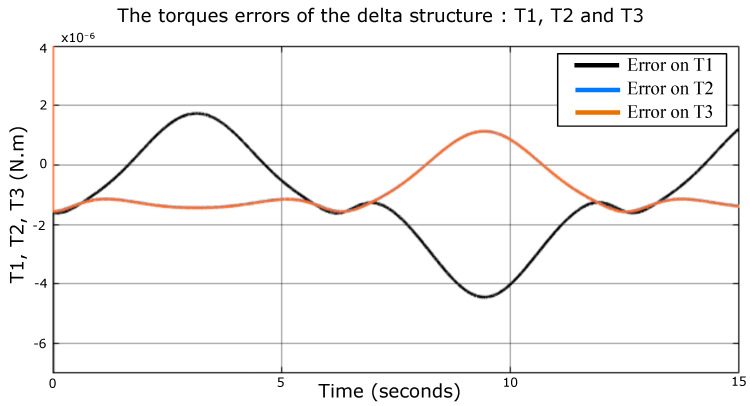
Torques errors signals of the Delta structure (the blue curve match the orange curve).

**Figure 27:**
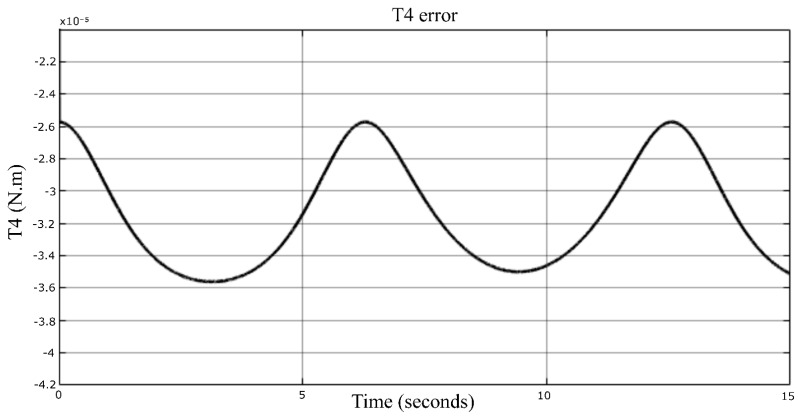
Torque error signal of the fourth actuator.

**Figure 28:**
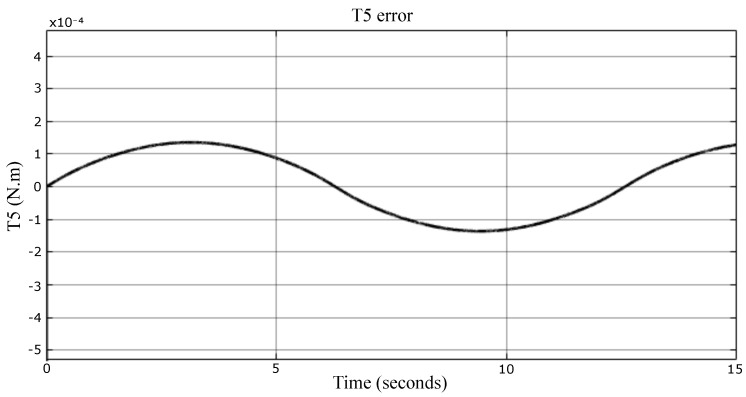
Torque error signal of the fifth actuator.

The dynamic model, Equation (35), is compared with the numerical model imported from the SolidWorks environment in this section, shown in Figure 25. In this simulation, the actuator moves in space along a desired trajectory, and its mass is 1 kg. The velocity is not as low as previously supposed. It is considerable enough to know the extent of the model accuracy. Figure 26, Figure 27 and Figure 28 represent the errors of the torques $T_{i}$. Even though the movement of the robot was fast, the reason to not calculate the different organs’ dynamics (due to their accelerations) is still fulfilled here. In fact, the simulation shows that the models have high accuracy, since the errors are $10^{-6}$, $10^{-5}$, and $10^{-4}$ N·m, which means there is no error.

## 7. The State Model

In this system, the state variables are the rotation angles $\theta_{i}$, described in Equation (34), their angular velocities ${\dot{\theta }}_{i}$, and the current of each motor $I_{i}$. The tensions $u_{1}$, $u_{2}$, $u_{3}$, $u_{4}$, and $u_{5}$ are the system inputs. The set of differential equations for each motor are

(36) $$\left\{\begin{array}{c} \dot{I_{i}}=-\frac{R}{L}\cdot I_{i}-\frac{K}{L}\cdot \dot{\theta }+\frac{1}{L}u_{i}\\ \ddot{\theta_{i}}=\frac{1}{J}\cdot T_{i}-\frac{1}{J}\cdot T_{ig}\\ T_{i}=K\cdot I_{i} \end{array}\right.$$

These equations represent, respectively, the electrical equation, the mechanical equation, and the electromechanical equation. *R* is the resistance, *L* is the induction, and *K* is the velocity constant (it is equal to the torque constant).

Let us take the following denotations:

$$a_{3}=\frac{K}{J},\;a_{g}=\frac{-1}{J},\;b_{1}=\frac{1}{L},\;b_{2}=\frac{-K}{L},\;b_{3}=\frac{-R}{L}.$$

and:

$$x_{1}=\theta_{i},\;x_{2}=\dot{\theta_{i}},\;x_{3}=I_{i}$$

Then, each motor state model will be as following:

(37) $$\left\{\begin{array}{c} \dot{x_{1}}=x_{2}\\ \dot{x_{2}}=a_{3}\cdot x_{3}+a_{g}\cdot T_{ig}\\ \dot{x_{3}}=b_{3}\cdot x_{3}+b_{2}\cdot x_{2}+b_{1}\cdot u_{i} \end{array}\right.$$

**Figure 29:**
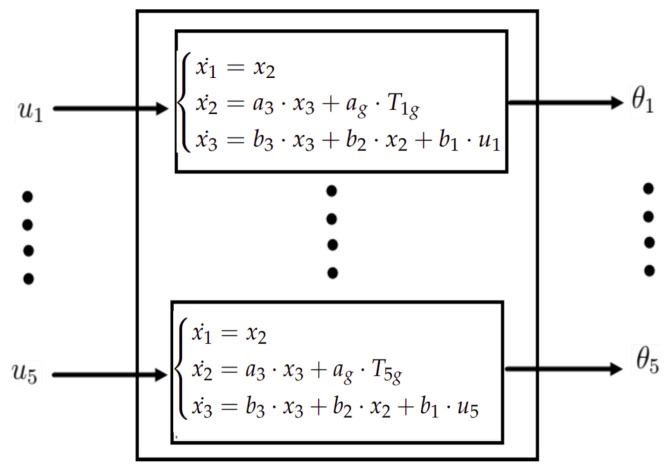
State model diagram.

The whole robotic system model is illustrated in Figure 29.

## 8. Controller

### 8.1. Control Architecture

**Figure 30:**
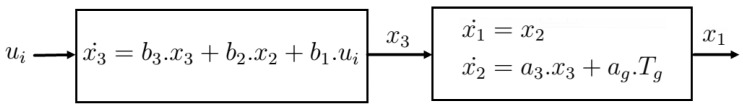
The electrical and mechanical subsystems.

This is a non-linear system of 15 state variables. A PID controller can be introduced if we consider $T_{ig}$ as a perturbation on the system. Alternatively, a better choice would be to transform the model to a linear one with feedback linearization. The state model can be separated to two submodels, as illustrated in Figure 30 below:

The electrical subsystem can be linearized and decoupled from the mechanical subsystem by applying feedback linearization $u_{i}=\frac{-b_{2}\cdot x_{2}+v_{i}}{b_{1}}$, which will undo the mechanical term to obtain the following linear submodel:

(38) $$\left\{\begin{array}{c} \dot{x_{3}}=b_{3}\cdot x_{3}+v_{i} \end{array}\right.$$

This linear system can be controlled with linear controllers such as a PID. We take advantage of the fact that the electrical subsystem is much faster then the mechanical subsystem, so we directly control the mechanical subsytem via the output of the electrical subsystem (cascade controller). We consider $x_{3}$ as the input of the mechanical subsystem.

We apply the linearizer feedback: $x_{3}=\frac{-a_{g}\cdot T_{g}+v}{a_{3}}$ to get a linear process:

(39) $$\left(\begin{array}{c} {\dot{x}}_{1}\\ {\dot{x}}_{2} \end{array}\right)=\left(\begin{array}{cc} 0 & 1\\ 0 & 0 \end{array}\right)\cdot \left(\begin{array}{c} x_{1}\\ x_{2} \end{array}\right)+\left(\begin{array}{c} 0\\ 1 \end{array}\right)\cdot v$$

This decoupled linearized system can be controlled with a linear controller. The friction of the various Delta structure parts will have almost no effect on the motors torques due to the following reasons:

1. As discussed and validated in the dynamic model section, the velocity and acceleration of the various Delta structures have no effect on the motors’ torques because of the high-reduction and the slowness required during the operation. Thus, the friction is also neglected since it is proportional to the velicty.
2. In case we want to consider friction of the motors rotors, the Equation (39) takes the following form:
  (40) $$\left(\begin{array}{c} {\dot{x}}_{1}\\ {\dot{x}}_{2} \end{array}\right)=\left(\begin{array}{cc} 0 & 1\\ 0 & -\zeta \end{array}\right)\cdot \left(\begin{array}{c} x_{1}\\ x_{2} \end{array}\right)+\left(\begin{array}{c} 0\\ 1 \end{array}\right)\cdot v$$
  With ($\zeta \cdot x_{2}$) being the friction of each motor.

**Figure 31:**
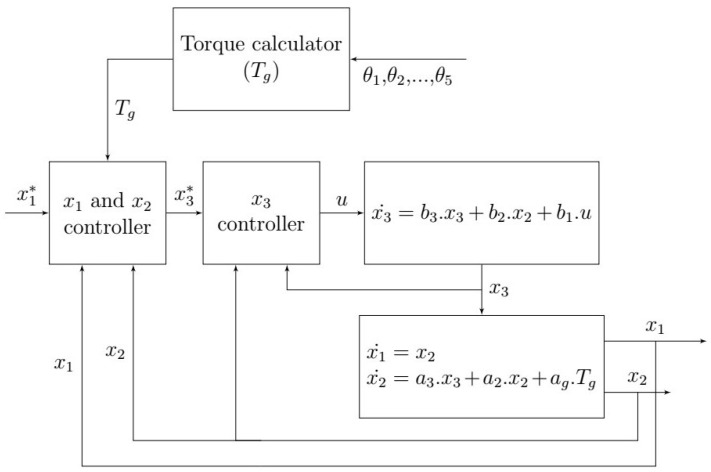
The synoptic diagram of the controller for each motor.

It is convenient to deploy a cascade control that involves two controllers, one of which nestles inside the other, such that the outer controller (position controller) feeds the inner one (current controller) with the set references, $x_{3}^{*}$, shown in Figure 31. Such a system can give an improved response to disturbances [44].

### 8.2. Trajectory, Validation by Simulation

In this section, we simulate the controller by assigning a desired trajectory to the end effector. The point $L_{2}$, that travels through the cochlea, is approximated to be a helical curve, on which the axis $L_{1}L_{2}$ must be tangent. The arc makes multiple half turns because of patient body constraints.

**Figure 32:**
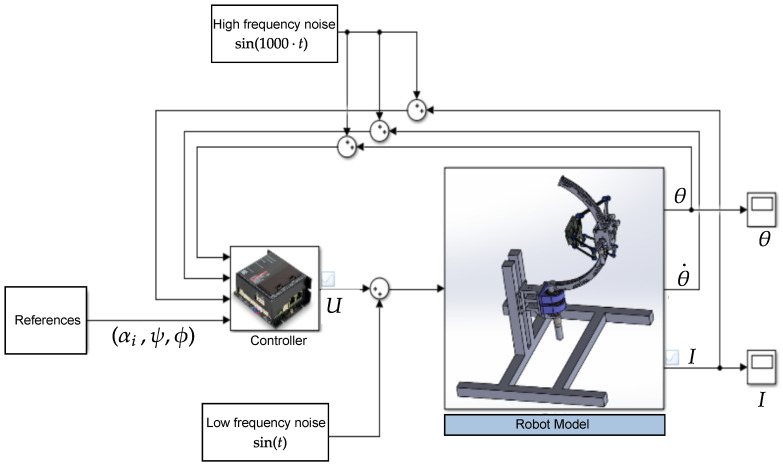
Simulink diagram to compare references and measurements of the angles.

**Figure 33:**
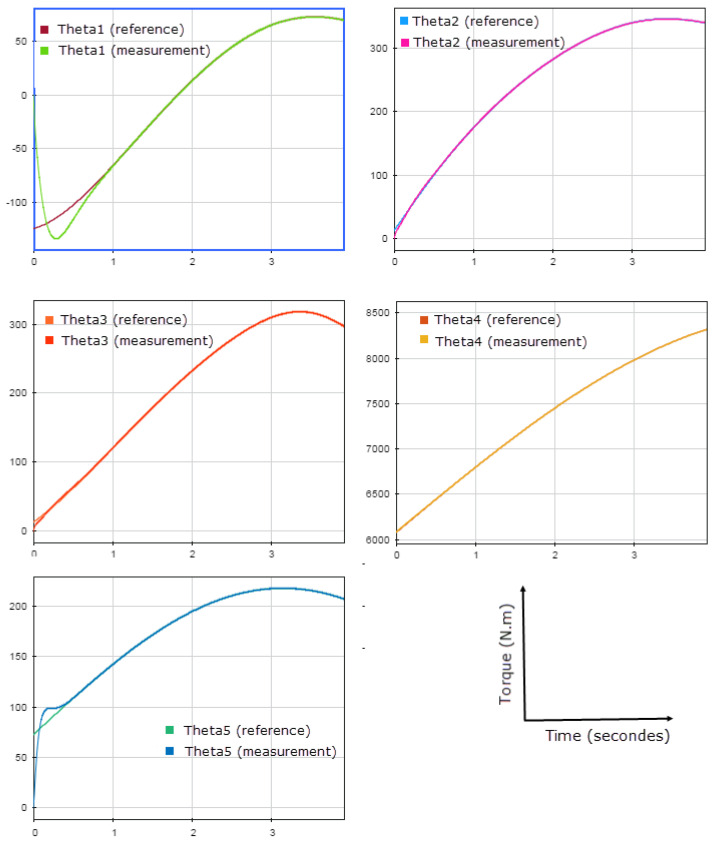
Comparison between the references and the measurements of the angles.

**Figure 34:**
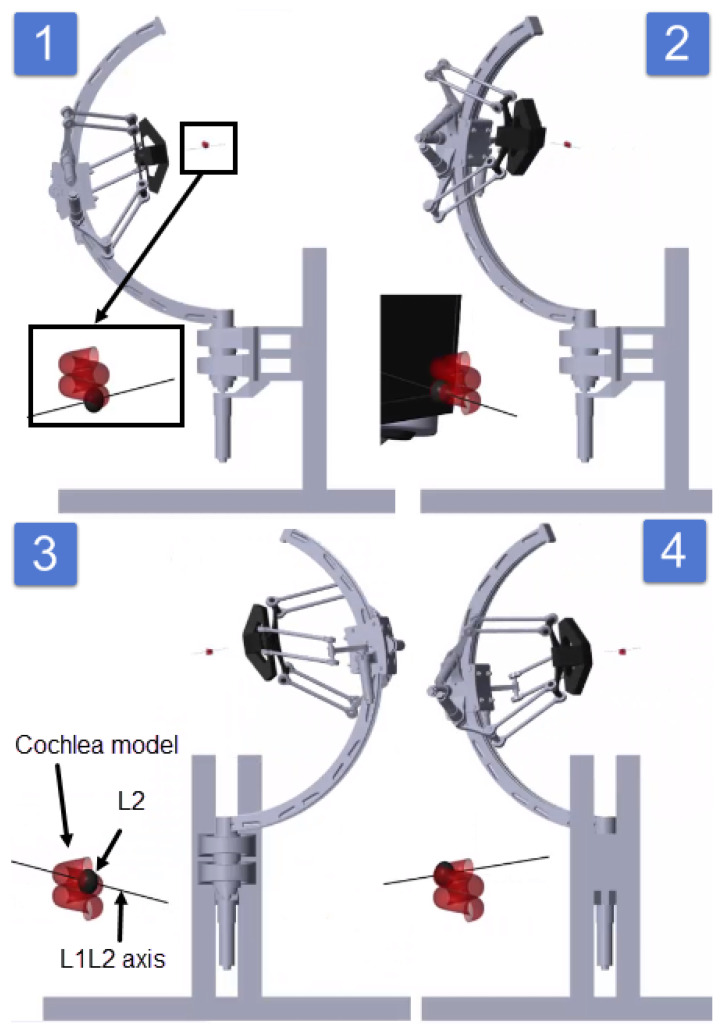
Snapshots (1–4) for the end effector travel through the cochlea.

The simulation, made using SimMechanics and shown in Figure 32, gives the results represented in Figure 33. Snapshots are shown in Figure 34.

The results illustrated in Figure 33 show that the motors’ angles very accurately follow the given references. Figure 34 shows that $L_{2}$ could travel through the desired trajectory, keeping the axis $L_{2}L_{1}$ tangent on the curve. This validates both the IKM and the controller.

## 9. Prototyping and Experimental Validation

**Figure 35:**
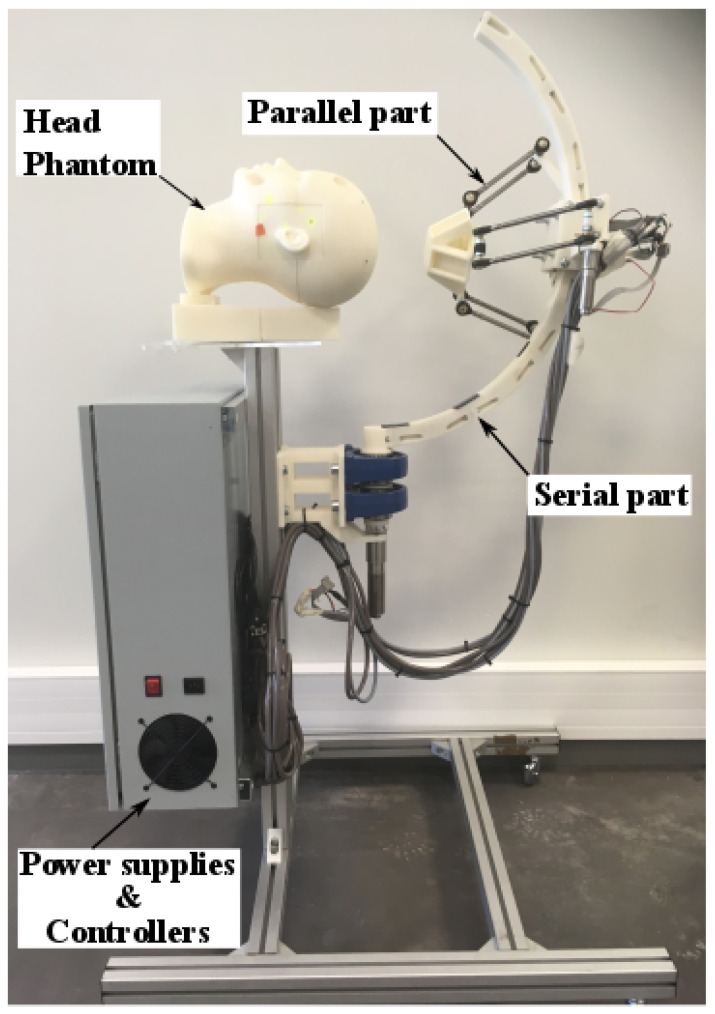
CochleRob manipulator prototype.

After manufacturing the various mechanical organs of the platform via an additive manufacturing technique, all these parts, including the sensors and actuators, have been assembled to make the first prototype of the CochleRob manipulator (Figure 35). This manipulator is controlled via a computer using LabVIEW software and Maxon controllers. The motors are Maxon as well. They were chosen after a trade-off was done between its length (which should ideally be short) and its torque (which should be higher). This implemented closed loop uses a classic PID controller.

**Figure 36:**
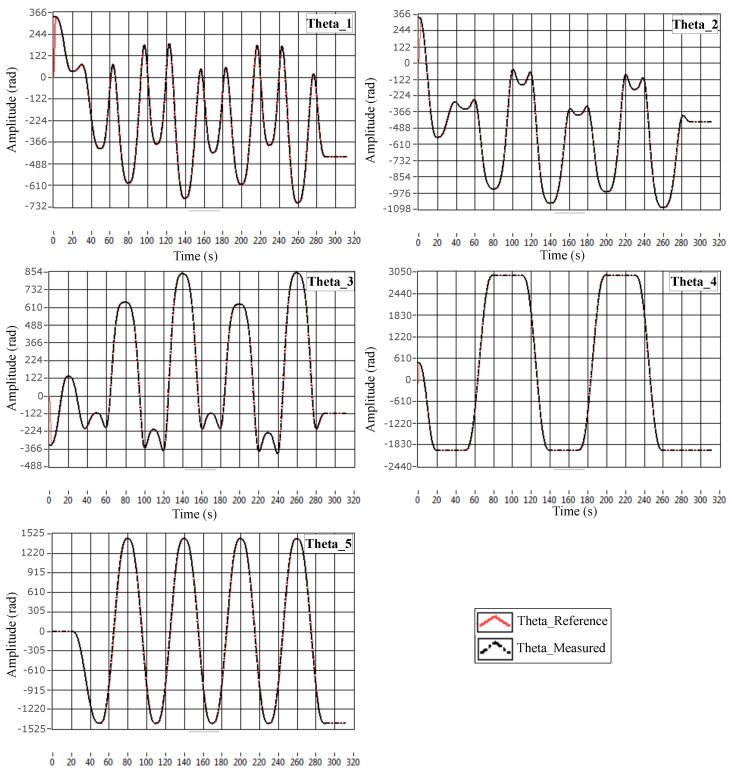
Experimental validation results.

The experimental results are illustrated in Figure 36. As we can see, the motors’ angles very accurately follow the given references. This experiment validates the operation of the platform and the different models developed in this work.

## 10. Conclusions and Perspectives

Our research into a new solution for treating inner ear diseases by remotely-controlled local drug administration resulted in a novel manipulator robot, CochleRob. The following are the key findings and achievements of our work:

- Our work introduces a new approach for intracochlear drug administration for the treatment of hearing loss through the development of the hybrid parallel–serial robot, CochleRob. This robot has a compact, precise, and rigid structure with five degrees of freedom that meets the requirements of positioning a magnetic actuator within the inner ear, including the necessary workspace and degrees of freedom.
- CochleRob reduces the risk of cochlear damage by the introduction of electrode arrays or catheters inside the cochlea. Drugs are delivered remotely, without the need for catheter or CI insertion.
- Through the validation of mathematical models, including kinematics, dynamics, and control laws, we have demonstrated the feasibility and effectiveness of our proposed solution. The results obtained from simulations were highly satisfactory, supporting the potential of CochleRob as a promising solution for the safe treatment of hearing loss.
- The proposed robot has been successfully prototyped and its components were manufactured using the additive manufacturing process. The robot was effectively controlled using Labview software, further demonstrating its viability as a solution for treating hearing loss. The motors chosen for the robot met the necessary specifications for torque and volume.

It is important to note that, while our proposed solution presents a promising step towards safe and effective treatment of hearing loss, further research and development are necessary to fully exploit its potential and to enhance the feasibility and reliability of our proposed robot. For example:

- It is important to consider the potential movement of the patient. Currently, it is possible to perform the procedure with a stabilized head using a face mask, a headband, or even anesthesia; however, this solution may not always be feasible or desirable. To address this issue, the head position can be tracked and fed back to the controller in real-time, enabling the adjustment of the assigned trajectory and reducing the risk of error. Several potential solutions for tracking the head position can be investigated, including deep neural network-based visual tracking using face landmark detection, among others.
- In order to expand the volume of the workspace and potentially increase compactness, it is worth conducting a thorough analysis of the workspace of the Delta structure based on its lengths, $l_{1}$, $l_{2}$, and *L* (the distance between $L_{2}$ and the nacelle center $P_{n}$). The distances, $l_{0}$ and $l_{3}$, are doomed to be invariable (or variable in a very short range) by the constraints on the motors’ lengths and the end-effector.

## Data Availability

Data are available from the corresponding author on request.

## Abbreviations

The following abbreviations are used in this manuscript:

CAD	Computer Aided Design
RWM	Round Window Membrane
CI	Cochlear Implant
SPMNP	SuperParamagnetic NanoParticles
LSC	Lateral Semicircular Canal
DoF	Degrees-of-Freedom
RCM	Remote Center of Motion
FKM	Forward Kinematics Model
IKM	Inverse Kinematics Model
PID	Proportional Integral Derivative
